# VPAC1 receptors play a dominant role in PACAP-induced vasorelaxation in female mice

**DOI:** 10.1371/journal.pone.0211433

**Published:** 2019-01-25

**Authors:** Ivan Ivic, Marta Balasko, Balazs D. Fulop, Hitoshi Hashimoto, Gabor Toth, Andrea Tamas, Tamas Juhasz, Akos Koller, Dora Reglodi, Margit Solymár

**Affiliations:** 1 Department of Anatomy, MTA-PTE PACAP Research Group, Medical School, University of Pecs, Pecs, Hungary; 2 Department of Physiology and Immunology, Medical School Osijek, University of J.J. Strossmayer, Osijek, Croatia; 3 Institute for Translational Medicine, Medical School, University of Pecs, Pecs, Hungary; 4 Laboratory of Molecular Neuropharmacology, Graduate School of Pharmaceutical Sciences, Osaka University, Suita, Osaka, Japan; 5 Molecular Research Center for Children’s Mental Development, United Graduate School of Child Development, Osaka University, Suita, Osaka, Japan; 6 Division of Bioscience, Institute for Datability Science, Osaka University, Suita, Osaka, Japan; 7 Department of Medical Chemistry, University of Szeged, Szeged, Hungary; 8 Department of Anatomy, Histology, and Embryology, Faculty of Medicine, University of Debrecen, Debrecen, Hungary; 9 Department of Physiology, New York Medical College, Valhalla, NY, United States of America; 10 Department of Neurosurgery, Medical School, University of Pecs, Pecs, Hungary; 11 Institute of Natural Sciences, Research Group for Sportgenetics and Sportgerontology, University of Physical Education, Budapest, Hungary; National University of Ireland Galway, IRELAND

## Abstract

**Background:**

PACAP and VIP are closely related neuropeptides with wide distribution and potent effect in the vasculature. We previously reported vasomotor activity in peripheral vasculature of male wild type (WT) and PACAP-deficient (KO) mice. However, female vascular responses are still unexplored. We hypothesized that PACAP-like activity is maintained in female PACAP KO mice and the mechanism through which it is regulated differs from that of male PACAP KO animals.

**Methods:**

We investigated the vasomotor effects of VIP and PACAP isoforms and their selective blockers in WT and PACAP KO female mice in carotid and femoral arteries. The expression and level of different PACAP receptors in the vessels were measured by RT-PCR and Western blot.

**Results:**

In both carotid and femoral arteries of WT mice, PACAP1-38, PACAP1-27 or VIP induced relaxation, without pronounced differences between them. Reduced relaxation was recorded only in the carotid arteries of KO mice as compared to their WT controls. The specific VPAC1R antagonist completely blocked the PACAP/VIP-induced relaxation in both arteries of all mice, while PAC1R antagonist affected relaxation only in their femoral arteries.

**Conclusion:**

In female WT mice, VPAC1 receptors appear to play a dominant role in PACAP-induced vasorelaxation both in carotid and in femoral arteries. In the PACAP KO group PAC1R activation exerts vasorelaxation in the femoral arteries but in carotid arteries there is no significant effect of the activation of this receptor. In the background of this regional difference, decreased PAC1R and increased VPAC1R availability in the carotid arteries was found.

## Introduction

Pituitary adenylate cyclase-activating polypeptide (PACAP) is a member of vasoactive intestinal peptide (VIP)/secretin/glucagon superfamily [1–3]. PACAP exists in two biologically active forms, PACAP1-38 and PACAP1-27, sharing the same N-terminal 27 amino acids. PACAP1-27 shares 68% structure identity with VIP. The structure of PACAP1-38 is well conserved through evolution, suggesting the involvement of this neuropeptide in a diverse array of biological functions in many tissues and organs [4–7]. Some of these diverse biological functions include angiogenic capacity and vascular smooth muscle (SM) relaxation [7, 8]. The actions of PACAP are mediated via the PAC1 receptor (PAC1R) which is specific for PACAP and through VPAC1/VPAC2 receptors (VPAC1R and VPAC2R) which bind both PACAP and VIP with similar affinity [7, 9–11]. All receptors belong to the group of G protein-coupled receptors (secretin receptor family). Expression of PACAP isoforms and their receptors has been reported in the central nervous system [7, 12, 13] and in peripheral organs, including blood vessels [7, 14].

Both PACAP and VIP have a potent effect on the vasculature, inducing relaxation and causing a decrease in local and systemic blood pressure [15, 16]. *In vitro* potent effect of PACAP in the vasculature has been demonstrated in various animal models, such as mice [17, 18], rats [19–21], cats [22, 23], dogs [19], pigs [16] and humans [7, 24].

This action is mediated through all three PACAP receptors localized mainly on the surface of the smooth muscle in arteries and arterioles [14, 25]. Although highly expressed, receptors are not detected equally across the vasculature. Receptors can be found in the small pulmonary arterioles and cerebral microvessels and also in large vessels like the aorta [7, 8, 14, 23, 26]. We have also confirmed the presence of PAC1R and VPAC1R in carotid and femoral artery of male mice [17].

Utilization of PACAP-deficient mouse model (knockout—KO) enables insight of physiological roles of PACAP both *in vivo* and *in vitro*. A thorough analysis of PACAP KO mice reported a number of different abnormalities resulting in several pathological alterations in different organs and higher mortality rate. Among others, PACAP deficiency leads to developmental alterations [27–29] and increased vulnerability to harmful stimuli as well as accelerated aging [30–32]. Our recent results show that PACAP deficiency leads to accelerated age-related systemic amyloidosis, with more severe and more generalized appearance of amyloid deposits in several organs in knockout mice [33].

The cardiovascular system is also affected by PACAP-deficiency. A recent study reported reduced dilatator ability of meningeal arteries upon nitroglycerin treatment in PACAP KO mice [18]. Another abnormality is related to the heart, where lack of PACAP leads to degeneration of the myocardium, increased fibrosis and reduced cardiac function [34, 35]. Our group reported increased arterial relaxation to PACAP1-27 and VIP and absence of relaxation to PACAP1-38 in male PACAP KO mice [17]. Although previously we did investigate how PACAP-deficiency influences vasomotor responses in males, such studies have not been conducted in females.

With regard to gender differences in various PACAP effects, the literature reported certain clinically important phenomena that differ in male and female groups. PACAP deficiency leads to decreased tear secretion and dry eye symptoms, which is more pronounced in female mice with aging [31]. The PACAP–PAC1 receptor pathway has been demonstrated to play a role in the human psychological stress responses. This study also revealed that in women the perturbations in the PACAP–PAC1 pathway are involved in abnormal stress responses [36]. Another group reported that a variant in the gene encoding PAC1 is associated with post-traumatic stress disorders in females [37] and later the role of estrogen was confirmed in this gender-dependent association [38]. As another significant clinical aspect, PACAP receptor emerged as a target in the therapy of migraine [39–41]. Migraine is one of the most common neurological disorders that is thought to be elicited by cerebral and meningeal arterial vasodilation [39–41]. Therefore, a thorough assessment of the vascular responses also in females is an important question for safe clinical use of such new drugs.

Our main question was whether the vascular response of isolated arteries to PACAP isoforms will be altered in female mice. Therefore, we aimed to investigate the relaxation properties of the carotid and femoral arteries of PACAP wild-type (WT) and KO female mice to cumulative administration of PACAP1-38, PACAP1-27 and VIP and in the presence of selective receptor blockers. We also aimed to investigate the presence of the various PACAP receptors in carotid and femoral arteries of female WT and PACAP KO mice.

## Materials and methods

### Animals

Experiments were performed on 8–12 week-old female PACAP-KO mice on a CD-1 background and their WT littermates (total n = 54). Our study has been approved by the University of Pecs Ethical Committee for the Protection of Animals in Research (BA02/2000-15024/2011). All procedures were in accordance with the main directives of the National Ethical Council for Animal Research and those of the European Communities Council (86/609/EEC, Directive 2010/63/EU of the European Parliament and of the Council).

### Surgery

The common carotid arteries and the proximal part of the femoral arteries were isolated using an Olympus surgical microscope (model SZX7; Olympus Inc., Japan) under anesthesia induced by intraperitoneal injection of ketamine (Gedeon Richter Plc., Budapest, Hungary) and xylazine (Eurovet Animal Health B.V., Bladel, Netherland) mixture (81.7 and 9.3 mg/kg, respectively). The proximal and distal ends of the isolated segment were ligated; the vessels were excised between the ligations, and then transferred to refrigerated Krebs solution. Carotid and femoral arteries of both sides were used. After the removal of the arteries, the animal was euthanized with an intraperitoneal injection of pentobarbital (100 mg/kg, Ceva Sante Animale, Libourna, France). Before every surgery, the female estrus cycle was tested by vaginal cytology [42].

### Measurement of isometric force of isolated arteries

Measurements of the isometric force of isolated arteries were according to Mulvany’s protocol and our previous experiments [17, 43]. After removal of the arteries, they were quickly transferred into cold oxygenated (95% O_2_/ 5% CO_2_) (Linde, Repcelak, Hungary) physiological Krebs solution (NaCl: 119 mM, KCl: 4.7 mM, KH_2_PO_4_: 1.2 mM, NaHCO_3_: 25 mM, Mg_2_SO_4_: 1.2 mM, CaCl_2_ × 2H_2_O: 1.6 mM, EDTA: 0.026 mM, glucose: 11.1 mM). NaCl and KCl were purchased from VWR International (Radnor, PA, USA). All other chemicals and drugs were obtained from Sigma-Aldrich (St. Louis, MO, USA). The arteries were dissected into 2 mm long rings. Each ring was positioned between two tungsten wires (diameter of the wire was 0.04 mm for CA and 0.02 mm for FA) in the organ chamber of the myograph in 5 ml Krebs bath solution. The bath solution was continuously oxygenated with a gas mixture of 95% O_2_ and 5% CO_2_, and kept at 36.9±0.1°C.

Isometric contraction forces were measured with a DMT 610M Wire Myograph (Danish Myo Technology, Aarhus, Denmark). Normalization was performed according to Mulvany and Harpern [43]. LabChart 8 (AD Instruments, Dunedin, New Zealand) and Myodaq 2.01 (Danish Myotechnologies, Denmark) software were used for data acquisition and display. After normalization, vessels were allowed to stabilize for 60 minutes.

### Pharmacological agents

At the beginning of the experiments, the functional integrity of the vessels was verified with viability tests. To study the endothelium-dependent [acetylcholine (Ach)-induced] and -independent [sodium nitroprusside (SNP)-induced] relaxation ability of the vessels first the vessels were pre-contracted with 60 mM KCl. When the contraction reached the plateau phase, 50 μl of increasing doses of either Ach or SNP were administered directly into the bath solution to reach final concentrations of 10^−9^ M to 10^−5^ M. After this viability test, the chambers were washed out with Krebs solution. Vascular responses of the vessels were measured in response to increasing doses of different vasoactive substances in a similar fashion. First, 60 mM KCl was administered to establish a tone [21, 44]. Once the vessel reached plateau phase, the pharmacological agent was applied directly into the vessel chamber to reach final concentration. After the administration of each dose of a specific substance, the isometric force was registered. We used PACAP1-38, PACAP1-27 and VIP with final concentrations of 10^-9^M to 10^-6^M (Bachem, Bubendorf, Switzerland). PACAP1-38 and PACAP1-27 were synthesized as previously described [45]. Selective agonists for PAC1R (maxadilan; Tocris, Bioscience, Bristol, UK), VPAC1R (Ala^11,22,28^VIP; Bachem) and VPAC2R (Bay55-9837; Bachem) were used (10^-10^M to 10^-7^M). In addition, antagonists for PAC1R/VPAC1R/VPAC2R (PACAP6-38, 10^-7^M), PAC1R (M65, 10^-7^M) and VPAC1R (VIP6-28, 10^-7^M) were also used (Bachem).

All drugs were dissolved in distilled water. When only the solvent (distilled water) was administrated, there was no change in force. Changes in the vasomotor activity were measured by the difference compared to maximal contraction induced by 60 mM KCl (in graphs marked as a baseline, 10^-0^M), for each administered drug, artery and mice genotype.

### RT-PCR analysis

Tissues were cryo-ground in liquid nitrogen and dissolved in Trizol (Applied Biosystems, Foster City, CA, USA), and after the addition of 20% RNase free chloroform samples were centrifuged at 4°C at 10,000×*g* for 15 min. Samples were incubated in 500 μL of RNase-free isopropanol at –20°C for 1 h then total RNA was harvested in RNase free water and stored at –20°C. The assay mixture for reverse transcriptase reaction contained 2 μg RNA, 0.112 μM oligo(dT), 0.5 mM dNTP, 200 units of High Capacity RT (Applied Bio-Systems) in 1× RT buffer. For the sequences of primer pairs and further details of polymerase chain reactions, see Table 1. Amplifications were performed in a thermal cycler (Labnet MultiGen 96-well Gradient Thermal Cycler; Labnet International, Edison, NJ, USA) in a final volume of 21 μL (containing 1 μL forward and reverse primers [0.4 μM], 0,5 μL dNTP [200 μM], and 5 units of Promega GoTaq DNA polymerase in 1× reaction buffer) as follows: 95°C, 2 min, followed by 35 cycles (denaturation, 94°C, 1 min; annealing at optimized temperatures as given in Table 1 for 1 min; extension, 72°C, 90 sec) and then 72°C, 10 min. PCR products were analyzed by electrophoresis in 1.2% agarose gel containing ethidium bromide. Actin was used as internal control. Signals were developed with gel documentary system (Fluorchem E, ProteinSimple, CA, USA). The optical density of signals was measured by using ImageJ 1.40g freeware and results were normalized to the optical density of control tissue.

**Table 1:** Nucleotide sequences, amplification sites, GenBank accession numbers, amplimer sizes and PCR reaction conditions for each primer pair are shown.

*Gene*	*Primer*	*Nucleotide sequence (5’→3’)*	*GenBank ID*	*Annealing temperature*	*Amplimer size (bp)*
	antisense	GCT GTA TTG CTC CTC CCT (518–535)			
**Actin** **(Actb)**	sense	GCC AAC CGT GAA AAG ATG A (419–437)	**NM_007393.5**	54°C	462
	antisense	CAA GAA GGA AGG CTG GAA AA (861–880)			
**PAC1 (ADCYAP1R1)**	sense	TAT TAC TAC CTG TCG GTG AAG (912–932)	**NM_016989.2**	49°C	213
	antisense	ATG ACT GCT GTC CTG CTC (1107–1124)			
**VPAC1** **(VIPR1)**	sense	TTT GAG GAT TTC GGG TGC (974–991)	**NM_001097523**	52°C	266
	antisense	TGG GCC TTA AAG TTG TCG (1222–1239)			
**VPAC2** **(VIPR2)**	sense	CTC CTG GTA GCC ATC CTT (805–822)	**NM_001014970**	48°C	149
	antisense	ATG CTG TGG TCG TTT GTG (936–953)			

### Western blot analysis

Isolated FA and CA were washed in physiological NaCl solution then collected in 100 μL of RIPA (Radio Immuno Precipitation Assay)-buffer (150 mM NaCl; 1.0% NP40, 0.5% sodium deoxycholate; 50 mM Tris, pH 8.0) for homogenization containing protease inhibitors (Aprotinin, 10 μg/mL), Benzamidine (5 mM), Leupeptin (10 μg/mL), Trypsine inhibitor (10 μg/mL), PMSF (1 mM), EDTA (5 mM), EGTA (1 mM), Na-Fluoride (8 mM), Na-orthovanadate (1 mM). Samples were stored at –70°C. Measurements were repeated 3 times for each vessel, isolated from WT and KO mice (n = 3/group). First artery samples were mechanically ground then the suspensions were sonicated by pulsing burst for 30 secs at 40 A (Cole-Parmer, Illinois, USA). Total tissue lysates were used for Western blotting. Samples for SDS-PAGE were prepared by the addition of Laemmli electrophoresis sample buffer (4% SDS, 10% 2-mercaptoethanol, 20% glycerol, 0.004% bromophenol blue, 0.125 M Tris HCl pH 6.8) to tissue lysates to set equal protein concentration of samples, and boiled for 10 min.

A total of 20 μg of protein was separated by 10% SDS-PAGE gel for detection of PAC1, VPAC1 and VPAC2. Proteins were transferred electrophoretically to nitrocellulose membranes. After blocking in 5% non-fat dry milk in PBST (phosphate buffered saline with 0.1% Tween 20; 20 mM Na_2_HPO_4_, 115 mM NaCl, pH 7.4), membranes were washed and exposed to the primary antibodies overnight at 4°C. Polyclonal anti-PAC1 antibody (Sigma-Aldrich, St. Louis, MO, USA) in 1:500, polyclonal anti-VPAC1 antibody (Alomone Labs., Jerusalem, Israel) in 1:1000, polyclonal anti-VPAC2 antibody (Abcam, Cambridge, UK) in 1:800 dilutions were used (Table 2). After washing three times for 10 min with PBST, membranes were incubated with the secondary anti-rabbit IgG antibody (Bio-Rad Laboratories, CA, USA) in 1:1500 dilution in PBST containing 1% non-fat dry milk for 2 h at room temperature. Signals were detected by enhanced chemiluminescence (Advansta Inc., Menlo Park, CA, USA) according to the instructions of the manufacturer. Actin was used as internal control. The optical density of signals was measured by using ImageJ 1.40g freeware and the results were normalized to the optical density of control tissue. Signals were developed with gel documentary system (Fluorchem E, ProteinSimple, CA, USA).

**Table 2:** Tables of antibodies used in the experiments.

*Antibody*	*Host animal*	*Dilution*	*Distributor*
**Anti-PAC1**	rabbit, polyclonal,	1:600	Sigma-Aldrich, St. Louis, MO, USA **SAB2900695**
**Anti-VPAC1**	rabbit, polyclonal,	1:800	Alomone Labs., Jerusalem, Israel **AVR-001**
**Anti-VPAC2**	rabbit, polyclonal,	1:600	Abcam, Camridge, UK **AB28624**
**Anti-Actin**	mouse, monoclonal,	1:10000	Sigma-Aldrich, St. Louis, MO, USA **A2228**

### Statistical analysis

All data represented here were collected in series, as single-point measurement, compared across genotypes and dose points by two-way ANOVA (post hoc–Tukey). For Western Blot analysis, the student *t-test* was used. Statistical analyses were performed using Sigma Plot 12.5 (Systat, Chicago, IL, USA). Significate difference value was set at *p* <0.05. The data are reported as mean ± SEM.

## Results

### Administration of PACAP1-38, PACAP1-27 and VIP leads to relaxation of carotid and femoral arteries in female mice

**Fig 1:**
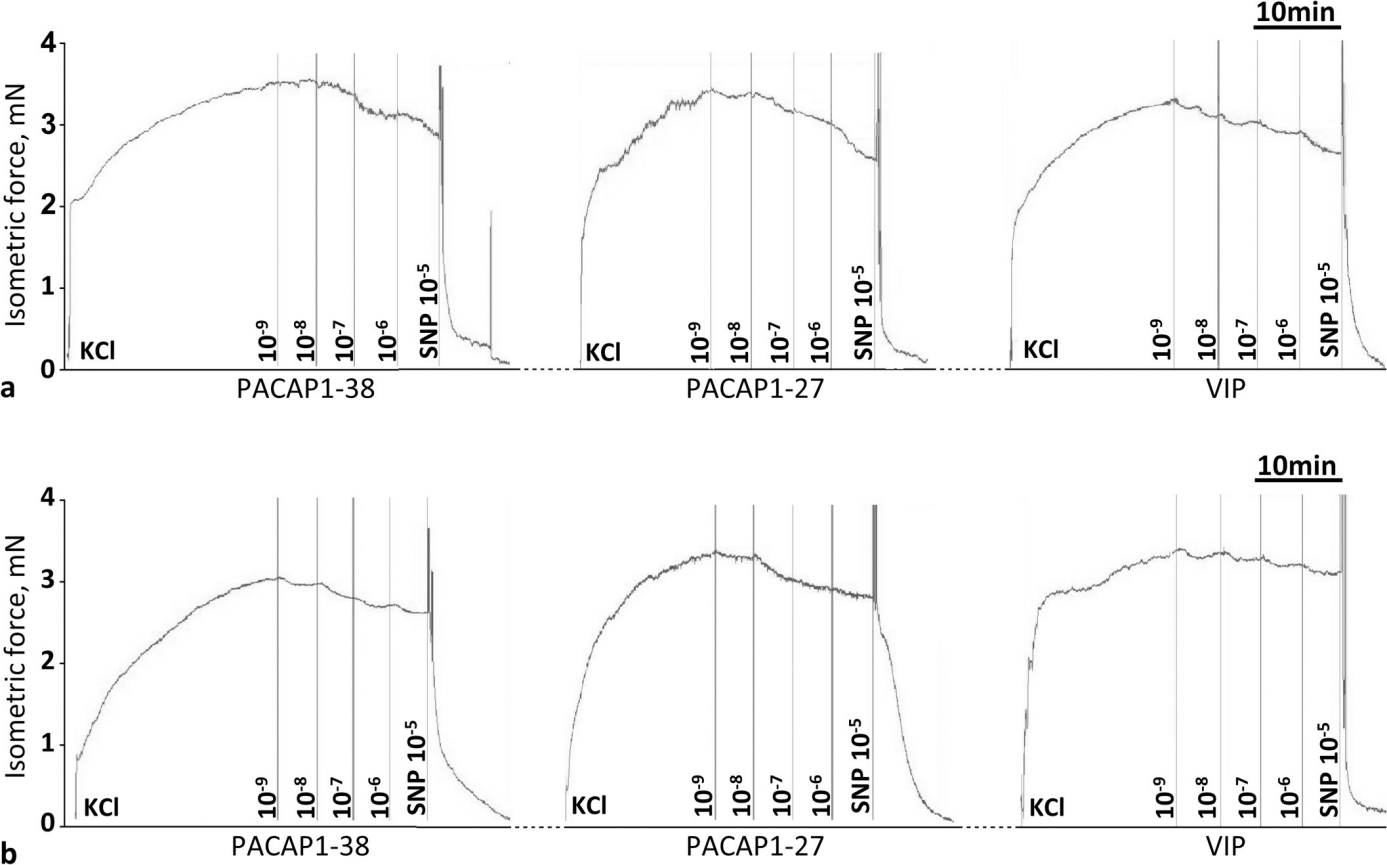
Original records show the cumulative dose-dependent effect of PACAP1–38, PACAP1-27 and VIP in the carotid artery of wild type (PACAP^+/+^, **a**) and PACAP KO (PACAP^-/-^, **b**) mice. At the end of the experiment, the viability of vessel was tested with sodium nitroprusside (SNP, 10^-5^M).

**Fig 2:**
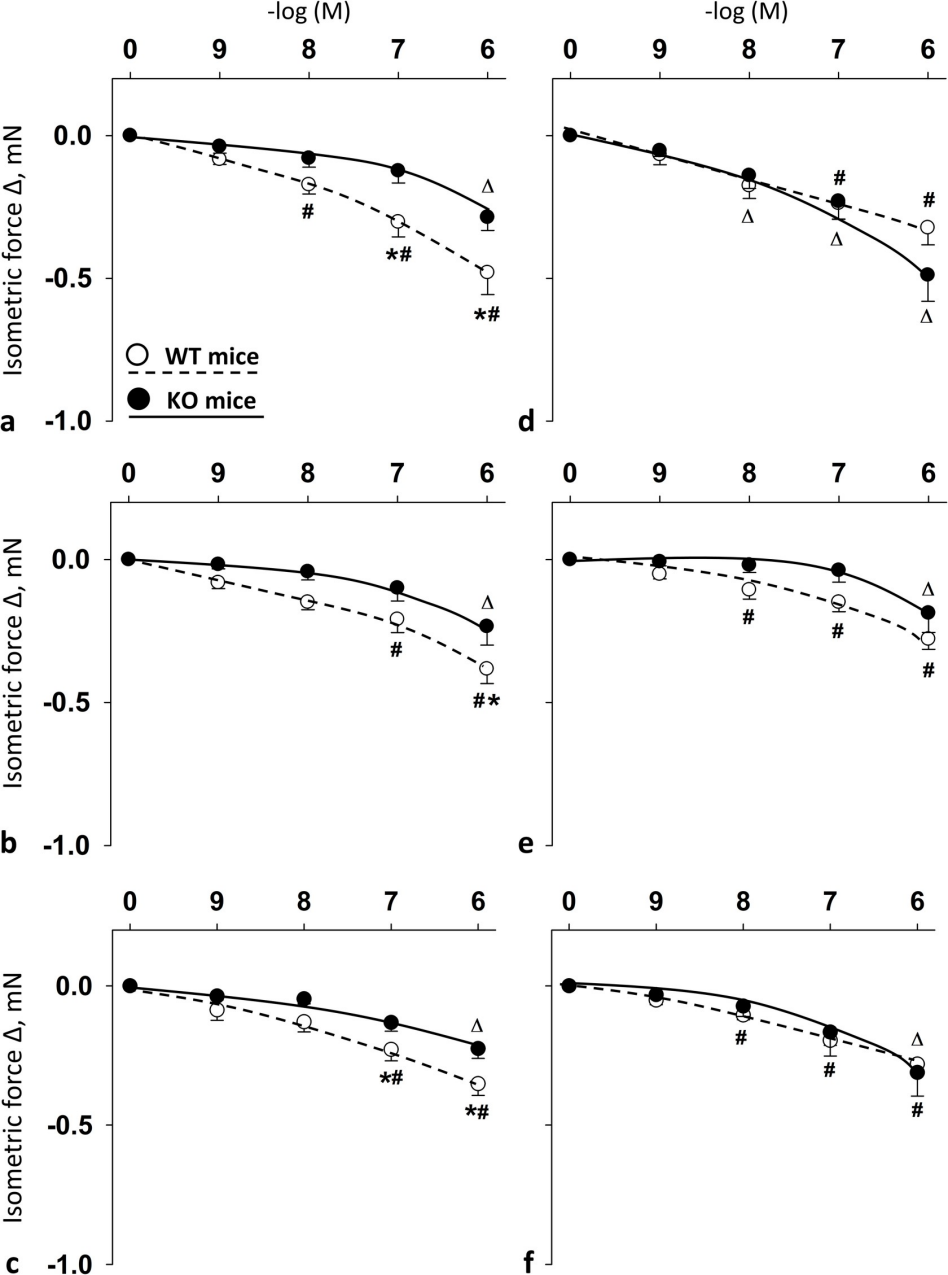
Vasomotor effect of cumulative dose-dependent administration of: PACAP1-38 (**a**); PACAP1-27 (**b**); and VIP (**c**) in carotid artery; and PACAP1-38 (**d**); PACAP1-27 (**e**); and VIP (**f**) in femoral artery of wild type (WT) mice and PACAP knockout (KO) mice. Arterial relaxation is marked as negative change in force. Data are expressed as means ± SEM (n = 6/group). *p < 0.05 WT vs. KO mice; ^#^*p* < 0.05 WT mice vs. baseline; ^Δ^*p* < 0.05 KO mice vs. baseline.

Original records (**Fig 1**) and summary data (**Fig 2A–2C**) show the effects of cumulative doses of PACAP1-38, PACAP1-27, and VIP on vasomotor responses of the isolated carotid arteries of WT and PACAP KO mice. With regard to WT mice, the presence of PACAP1-38 (10^−8^–10^-6^M), PACAP1-27 (10^−7^–10^-6^M) and VIP (10^−7^–10^-6^M) resulted in a significant dose-dependent relaxation. In contrast, in PACAP KO mice, administration of PACAP1-38, PACAP1-27 and VIP resulted in a reduced dose-dependent relaxation (only significant at 10^-6^M for each substance). There was a significant difference between the relaxation of WT and PACAP KO mice for each substance, i.e. for PACAP1-38 and VIP at 10^−7^–10^-6^M, and for PACAP1-27 at 10^-6^M.

Concerning femoral arteries of WT mice, PACAP1-38 (10^−7^–10^-6^M), PACAP1-27 and VIP (10^−8^–10^-6^M) induced significant relaxations. In PACAP KO mice, the presence of PACAP1-38 induced femoral relaxation at 10^−8^–10^-6^M, however PACAP1-27 and VIP only at 10^-6^M. In these arteries, there was no difference between vasomotor responses of WT and PACAP KO mice for any of the substances (**Fig 2D–2F**). The female estrus cycle did not influence these vasomotor responses of the arteries (**S4 Fig**).

As compared with females, PACAP- and VIP-induced arterial relaxations were significantly stronger both in male WT and in male PACAP KO mice as shown by **S1 Fig**.

### Selective PAC1R antagonist M65 blocked PACAP1-38-, PACAP1-27- and VIP-induced relaxation in femoral but not in carotid arteries in female mice

**Fig 3:**
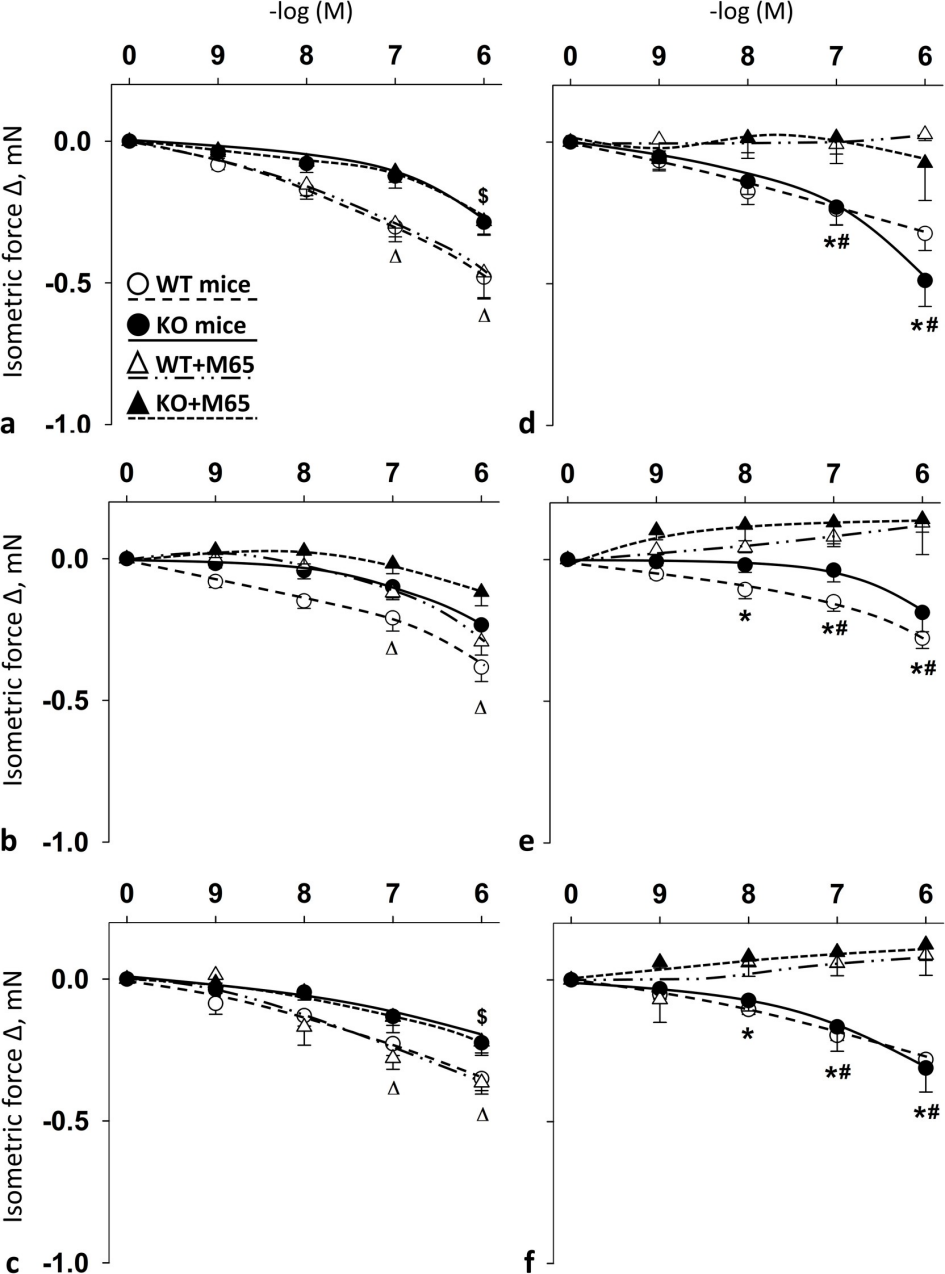
Vasomotor effect of cumulative dose-dependent administration of: PACAP1-38 (**a**), PACAP1-27 (**b**) and VIP (**c**) in carotid artery; and PACAP1-38 (**d**), PACAP1-27 (**e**) and VIP (**f**) in femoral artery of wild type (WT) and PACAP knockout (KO) mice in the presence of PAC1R antagonist M65. Arterial relaxation is marked as negative change in force. Data are expressed as means ± SEM (n = 3-6/group). *p < 0.05 WT vs. WT+M65 mice; ^#^*p* < 0.05 KO vs. KO+M65 mice; ^Δ^*p* < 0.05 WT+M65 vs. baseline; ^$^*p* < 0.05 KO+M65 vs. baseline.

Summary data show the effects of cumulative doses of PACAP1-38, PACAP1-27 and VIP with or without the presence of PAC1R antagonist M65 on the vasomotor responses in the carotid (**Fig 3A**–3**C**) and femoral arteries (**Fig 3D–3F**) of WT and PACAP KO mice. In the carotid arteries of both WT and PACAP KO mice, the presence of M65 had no significant impact on PACAP- and VIP-induced responses (**Fig 3A–3C**). However, in femoral arteries, the administration of M65 significantly reduced PACAP- and VIP-induced relaxation in both WT and PACAP KO mice at 10^−7^–10^-6^M (for PACAP1-27 and VIP in WT mice already at 10^-8^M) (**Fig 3D and 3E)**.

### Selective VPAC1R antagonist VIP6-28 blocked PACAP1-38-, PACAP1-27- and VIP-induced relaxation in both femoral and carotid arteries in female mice

**Fig 4:**
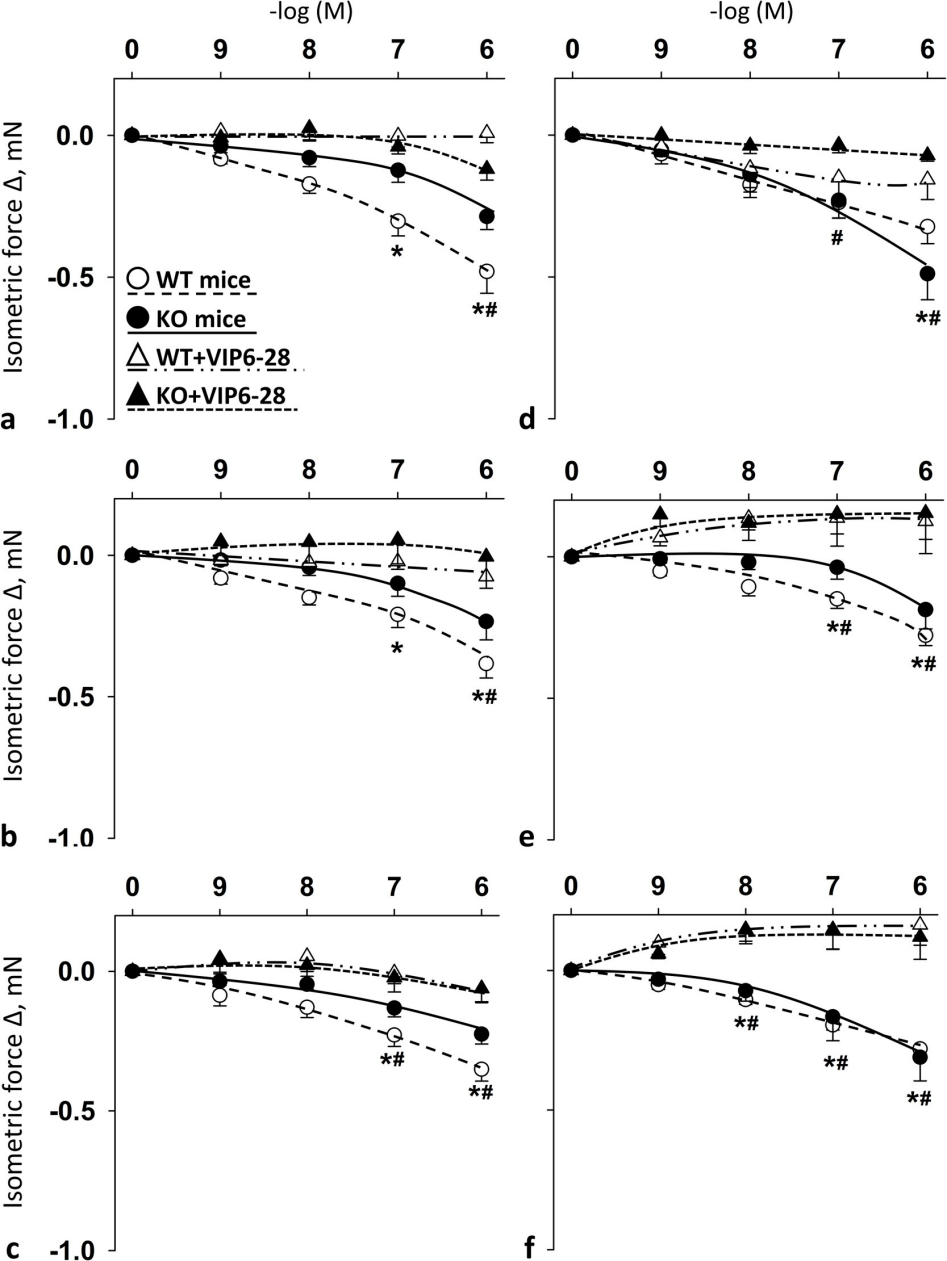
Vasomotor effect of cumulative dose-dependent administration of: PACAP1-38 (**a**), PACAP1-27 (**b**) and VIP (**c**) in carotid artery; and PACAP1-38 (**d**), PACAP1-27 (**e**) and VIP (**f**) in femoral artery of wild-type (WT) and PACAP knockout (KO) mice in the presence of VPAC1R antagonist VIP6-28. Arterial relaxation is marked as negative change in force. Data are expressed as means ± SEM (n = 3-6/group). *p < 0.05 WT vs. WT+VIP6-28 mice; ^#^*p* < 0.05 KO vs. KO+VIP6-28 mice.

**Fig 4** shows the vasomotor effects of cumulative doses of PACAP1-38, PACAP1-27 and VIP in the presence of VPAC1R antagonist VIP6-28 in carotid (**Fig 4A–4C**) and femoral arteries (**Fig 4D–4F**) of WT and PACAP KO mice. In the arteries of both WT and KO mice, the presence of VIP6-28 resulted in blockade of PACAP- and VIP-induced vasomotor responses as compared to their controls (**Fig 4A–4F**). The difference was always significant at 10^-6^M in WT and KO mice in both arteries for all examined substances. For additional significant differences see **Fig 4**.

### Selective PAC1R agonist maxadilan, VPAC1R agonist Ala^11,22,28^VIP, but not VPAC2R agonist Bay55-9837 induced relaxation in female mice

**Fig 5:**
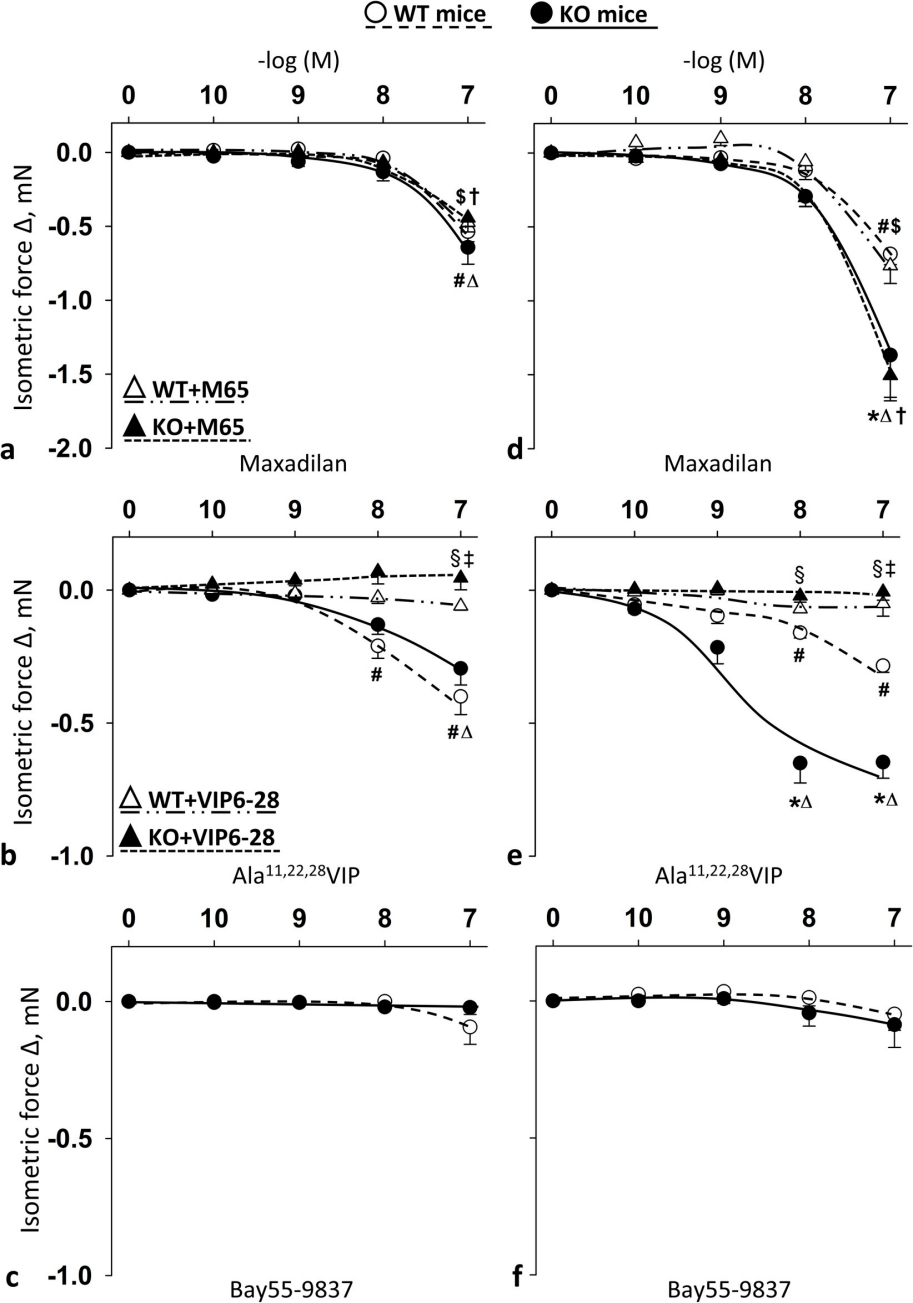
Vasomotor effect of cumulative dose-dependent administration of: Maxadilan (**a**); Ala^11,22,28^VIP (**b**); and Bay55-9837 (**c**) in carotid artery; and Maxadilan (**d**); Ala^11,22,28^VIP (**e**); and Bay55-9837 (**f**) in femoral artery of wild type (WT) and PACAP knockout (KO) mice, and their effect in the presence of M65 (PAC1R antagonist) (**a** and **d**) and VIP6-28 (VPAC1R antagonist) (**b** and **e**). Arterial relaxation is marked as negative change in force. Data are expressed as means ± SEM (n = 3-6/group). *p < 0.05 WT vs. KO; ^#^*p* < 0.05 WT vs. baseline; ^Δ^*p* < 0.05 KO vs. baseline; ^$^*p* < 0.05 WT+antagonist vs. baseline; †*p* < 0.05 KO+antagonist vs. baseline; ^**‡**^*p* < 0.05 WT vs. WT+antagonist; ^**§**^*p* < 0.05 KO vs. KO+antagonist.

Summary data show the effects of cumulative doses of receptor agonists in the carotid (**Fig 5A–5C**) and femoral arteries (**Fig 5D–5F**) of WT and PACAP KO mice with or without their respective selective blockers. Selective PAC1R agonist maxadilan induced significant relaxations of carotid and femoral arteries only at the highest concentration (10^-7^M) (**Fig 5A** and **5D**). PAC1R effect was remarkable stronger on femoral arteries of PACAP KO mice at this concentration compared to relaxation effect of WT mice **(Fig 5D)**. However, selective PAC1R antagonist M65 had no effect on the strong femoral relaxation of PACAP KO mice (**Fig 5A** and **5D**).

Selective VPAC1 agonist Ala^11,22,28^VIP elicited significant relaxation in femoral arteries of both WT and PACAP KO mice at 10^−8^–10^-7^M dose (on carotid arteries of PACAP KO mice only at 10^-7^M) (**Fig 5B** and **5E**). There was a significant difference between WT and PACAP KO mice in the femoral arteries (10^−8^–10^-7^M), where the selective VPAC1 agonist Ala^11,22,28^VIP induced greater relaxations in the PACAP KO mice compared to WT mice. The selective VPAC1R antagonist VIP6-28 completely inhibited all the above mentioned VPAC1R agonist Ala^11,22,28^VIP-induced relaxations.

On the other hand, selective VPAC2R agonist Bay55-9837 had no significant effect on vasomotor responses (**Fig 5C** and **5F**).

### Arteries of female PACAP KO mice express increased amount of VPAC1R and decreased amount of PAC1R compared to those of WT mice

**Fig 6:**
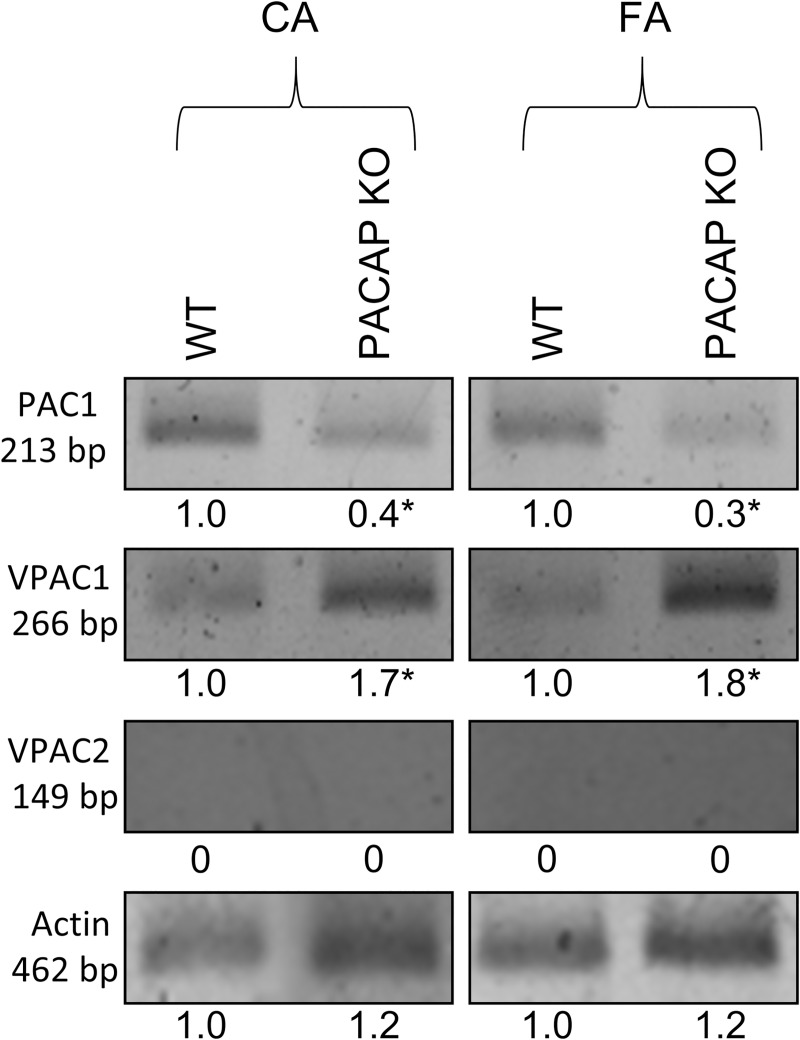
mRNA expression of PAC1R, VPAC1R and VPAC2R in femoral artery (FA) and carotid artery (CA) of wild type (WT) and PACAP knockout (KO) mice. Actin was used as an internal control. Numbers below signals represent integrated densities of signals determined by ImageJ software. mRNA expression of representative data of two independent animal samples. *p < 0.05 vs. control (WT mice).

**Fig 6** shows the mRNA expressions of PAC1R, VPAC1R, and VPAC2R detected by RT-PCR in carotid and femoral arteries of WT and PACAP KO mice. The analysis showed reduced expression of PAC1R and increased expression of VPAC1R in the arteries of PACAP KO mice as compared to WT controls (t-test, * p < 0.05 vs. control) (**Fig 6**). No VPAC2R expression was detected in either sample.

Protein levels analyzed by Western blot were in accord with the expression findings (t-test, * p < 0.05 vs. control). No signal for PAC1R protein was detected in carotid arteries of PACAP KO mice (**S2 Fig**).

### Arterial viability tests

In both types of arteries of PACAP KO mice, a 60mM KCl elicited significantly greater contractions as compared to their WT controls (**S3A Fig**).

The magnitudes of ACh- (endothelium dependent) and SNP-induced (endothelium independent) arterial relaxations were similar in WT and PACAP KO mice. The SNP-induced responses in femoral arteries were stronger compared to those of carotid arteries (**S3B** and **S3C Fig**).

### Non-selective PAC1R/VPAC1R/VPAC2R antagonist PACAP6-38 blocked vasomotor effects of PACAP1-38-, PACAP1-27- or VIP

Cumulative doses of PACAP1-38, PACAP1-27 and VIP failed to elicit any vasorelaxation in the arteries of female WT and PACAP KO mice in the presence of non-selective PAC1R/VPAC1R/VPAC2R antagonist PACAP6-38.

## Discussion

Our results showed that arteries of female mice reacted to administration of PACAP/VIP with vasorelaxation, similar to our previous observations in male mice [17]. However, vessels of female mice show weaker relaxations compared to males. Females also seem to be less sensitive to the lack of PACAP than males. The main novel finding of the current study is, that in female mice, VPAC1 receptors appear to play a dominant role in PACAP-induced vasorelaxation both in carotid and in femoral arteries. While in the femoral arteries PAC1R activation exerts vasorelaxation, in carotid arteries of PACAP KO mice, there is no significant effect of the activation of this receptor.

These findings are supported by multiple lines of evidence: 1) functional experiments show, that a selective VPAC1R antagonist is able to decrease the vasomotor effect of all PACAP isoforms both in carotid and in femoral arteries in both WT and PACAP KO mice; 2) both RT-PCR and the Western blot verify the VPAC1R upregulation together with the reduction of PAC1R mRNA and protein levels in PACAP KO mice.

### Vascular effect of PACAP isoforms and VIP in arteries isolated from female PACAP WT and KO mice

Potent vasomotor effect of PACAP has been described by many previous studies both *in vivo* and *in vitro* [7, 15, 16, 19, 35, 46, 47]. Various studies demonstrated relaxation responses to both PACAP isoforms and VIP in vessels of different origin, such as pulmonary [22], coronary [48], carotid [17, 21], cerebral and intracerebral [19, 48] of various species. In accordance with these studies, we have also observed arterial relaxation induced by PACAP/VIP administration in female mice. There was no difference between the magnitude of relaxation triggered by PACAP isoforms or VIP, in carotid and femoral arteries of WT mice. However, Cheng et al. reported the strongest vasomotor effect of PACAP1-27 in pulmonary arteries [22], whereas other studies reported the strongest vasodilator capacity of PACAP1-38 in rat mesenteric [49] and mice carotid and femoral arteries [17], suggesting isoform- and region-specific PACAP responses. Further studies confirmed such region-specific vascular effects of PACAP. In these studies, the same stimulus induced different magnitude of response in different organs [21], or even reacted differently within different regions of the same organ, e.g. brain vessels [14], probably reflecting different requirements of blood supply. The reason for this could be due to the different sensitivity of arteries to PACAP isoforms [50], and potency of receptors [7, 14].

Introduction of the PACAP KO model allows us to investigate these mechanisms further. While relaxations of carotid arteries to PACAP isoforms were reduced in female PACAP KO mice compared to WT controls to some extent, femoral arteries did not show such a difference.

### Involvement of PAC1, VPAC1 and VPAC2 receptors in vascular responses of female mice

As described earlier, PACAP/VIP receptor distribution can highly differ across and within organs [7]. Vasculature of animals and humans are innervated with PACAP-containing nerve fibers and as a result, they contain a high density of binding sites [7, 14, 20, 24, 51]. Our previous study also reported the presence of PAC1R and VPAC1R in peripheral arteries of mice [17], however, the presence of PAC1, VPAC1 and VPAC2 receptors in peripheral arteries of female mice have not been investigated yet.

According to our results, in the vasculature of female mice, presence and function of PAC1R was verified with the selective PAC1R agonist maxadilan. Interestingly, although PAC1 protein is either absent (in carotid arteries) or reduced (in femoral arteries) in PACAP KO mice, we observed relaxation in all arteries of both WT and PACAP KO mice after maxadilan treatment. We found similar observations in male mice [17]. PAC1R may be present in reduced amount in the vasculature of PACAP KO mice, which is not detectable with Western blot method. Selective antagonist of PAC1R had no effect on maxadilan-induced vasodilation, most likely due to the massive potency of maxadilan as compared with that of M65 or the existence of PAC1R splice variants [7] resulting in different affinity for maxadilan and M65. The idea that maxadilan is not selective enough should not be ignored, especially because it is not structurally related to PACAP/VIP. Interestingly, administration of M65 reduced PACAP/VIP-induced relaxation in femoral arteries, but not in carotid arteries. We propose that this difference in the effectivity of the pharmacological agents could be due to different downstream signaling response, a mechanism shown by Hoover et al. in cardiac neurons [52]. However, these suggested mechanisms still cannot fully explain the surprising fact that M65, as a maxadilan derivative fails to block the vasomotor effect of maxadilan.

Involvement of VPAC1R in arterial relaxation in female mice was confirmed with selective receptor agonist VIP6-28. VPAC1R is functional in both arteries of WT and PACAP KO female mice. This is consistent with other previous findings [7, 53] and with our previous work [17]. Our molecular analysis was in accord with the functional vasomotor responses of the arteries. VPAC1R agonist resulted in higher relaxation of femoral arteries in PACAP KO than in the WT mice. A possible explanation could be that endogenous PACAP suppresses receptor activity in benefit of PAC1R, and in the absence of PACAP, the signal transduction could shift toward VPAC1R. This is supported by the fact that PACAP upregulates the receptor expression [54], which can be altered by lack of PACAP. In addition, there is a possibility that in the absence of PACAP, the VPAC1R may undergo conformational changes allowing the binding of VPAC1R agonist Ala^11,22,28^VIP more effectively, despite a similar receptor expression. It could be the reason for an increased response to a selective agonist in the femoral arteries of KO mice. In contrast, there was no difference in the vasomotor responses of the carotid arteries of the WT and PACAP KO mice, which again may indicate the importance of region-specific vasomotor responses as mentioned above. Importantly, selective VPAC1R antagonist VIP6-28 abolished all PACAP- induced relaxations that suggests a crucial role of VPAC1R in the vasculature of female mice.

**Fig 7:**
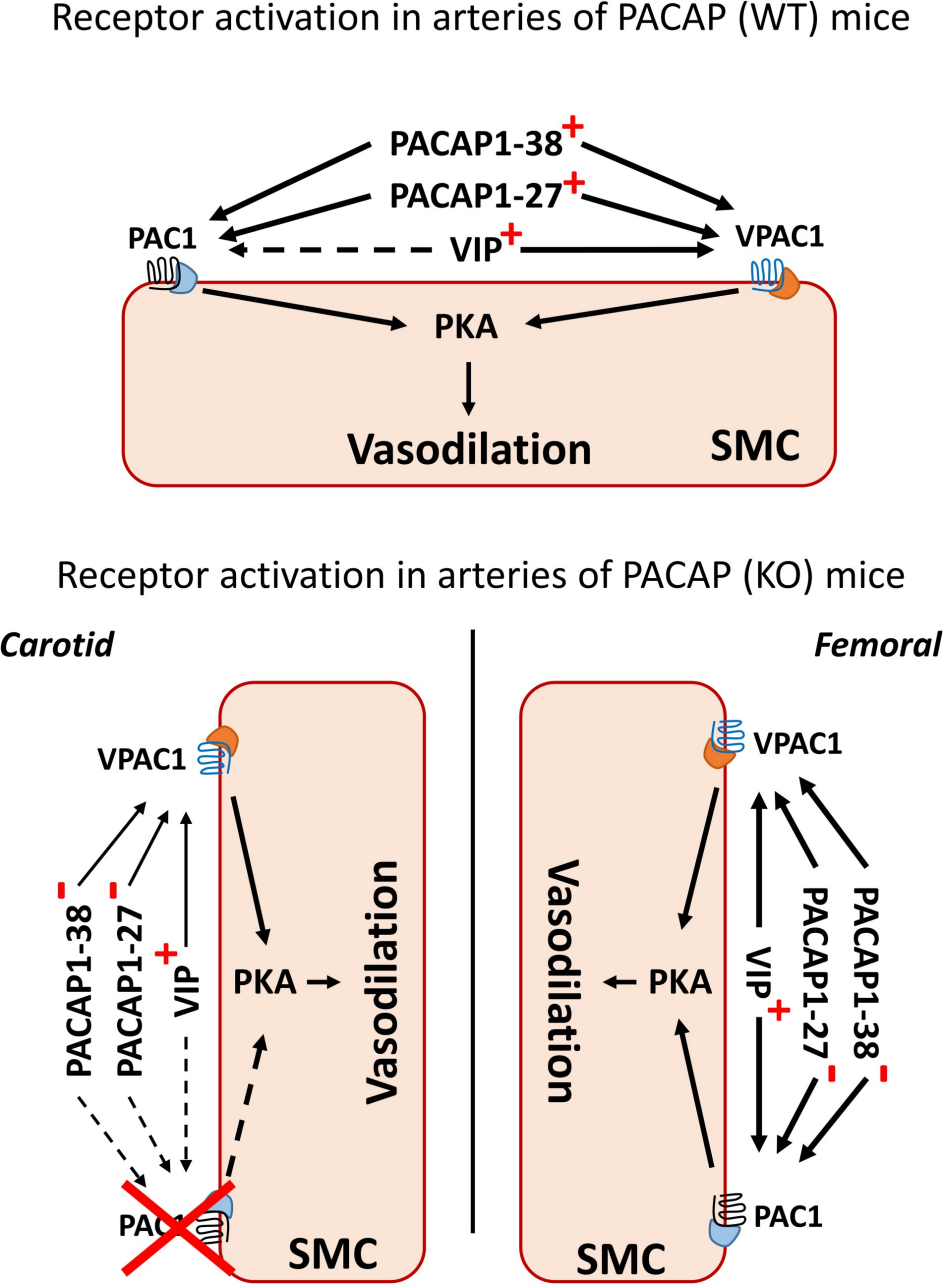
Proposed mechanisms of PACAP-induced relaxation in PACAP WT and KO female mice. **Upper panel:** PACAP and VIP bind to PAC1R/VPAC1R G protein-coupled receptors, and stimulate the cAMP/PKA, promoting vasodilatation. PACAP isoforms bind to both receptors, while VIP binds only to VPAC1R (and PAC1R at >500nM [7]). PACAP isoforms and VIP induce similar relaxations in arteries of WT and KO mice. **Lower panel:** in carotid arteries of PACAP-deficient mice, PACAP- and VIP-induced relaxations were reduced, whereas in femoral arteries there is no difference in response as compared to WT mice. Relaxation of carotid arteries is modulated primarily via VPAC1R and relaxation of femoral arteries via both PAC1R and VPAC1R. Endogenous VIP can bind not only to VPAC1R, but also to PAC1R. Unchanged, reduced or absent relaxations to polypeptides are marked with thicker, thinner or dashed arrows, respectively (as compared to the PACAP wild type mice). “**+**” indicates polypeptides presented in the system and added exogenously. “**-**”indicates exogenously added polypeptides (in PACAP-deficient mice only).

Observations with PACAP isoforms and VIP in the presence of selective PAC1R (M65) and VPAC1R (Ala_11,22,28_VIP) antagonists indicates that receptor signaling is different in carotid and femoral arteries. It seems that in carotid arteries, only VPAC1R is functional, while in femoral arteries synergistical activation of both PAC1R and VPAC1R allows a vascular response (**Fig 7**). Although molecular findings show some inconsistency with functional findings (effect of PAC1R agonist in carotid arteries), nevertheless, these findings indicate the importance of VPAC1R in the modulation of response not only in female WT [14] but also in PACAP KO mice.

### Gender difference in PACAP- and VIP-induced relaxation in arteries of mice

Vessels of female mice show weaker vascular relaxations upon PACAP isoforms and VIP administration as compared to those of males [17]. Females also seem to be less sensitive to the lack of PACAP than males. Other peptides, like endothelin-1 and bradykinin (femoral and brachial arteries) [55] or neuropeptide Y (hind limb) [56] also show gender-dependent effects in the vasculature experimental animals. Different receptor modulation and downstream signaling in females could play a role in the observed gender- differences of vascular responses, as we can see from our experiments with selective antagonists.

The question arises, whether sex steroids affect PACAP/VIP-induced relaxation. However, there were no significant differences in PACAP- and VIP-induced vascular responses during the estrus cycle. Other observations reported by Dalsgaard and coworkers also showed that treatment with sex steroids induced no changes in the vascular effects of PACAP or VIP in ovariectomized rabbits [48]. Gender-difference was also reported with regard to other effects of PACAP or VIP. Kiss and coworkers demonstrated a marked increase in PACAP levels in the hypothalamus of male rats after food deprivation compared to the lower increase in females [57], while Lam and coworkers reported higher levels of VIP were found in the pituitaries of male as compared to females. Although these studies demonstrate PACAP/VIP related gender-differences [57, 58], relatively little is known about differences in either expression of receptors or effect of PACAP in the macrovasculature. Further investigations in ovariectomized female mice would help clarify the underlying mechanisms.

## Conclusions

This study is the first to show the relaxation effects of exogenous PACAP isoforms in peripheral arteries isolated from female WT and PACAP-deficient mice. These responses are more moderate than those of male mice. In female PACAP KO mice, carotid vasomotor responses to PACAP isoforms and VIP were reduced, but no differences were detected in the femoral arteries. In the background of this regional difference, decreased PAC1R and increased VPAC1R mediation of the carotid arteries were found. Our results also show, that in female mice, PACAP-induced vasorelaxation responses are mainly mediated by VPAC1R.

## Supporting information

S1 Fig Gender-dependent differences of cumulative dose-dependent administration of PACAP1-38 (**a** and **d**); PACAP1-27 (**b** and **e**) and VIP (**c** and **f**) on the vasomotor response in carotid arteries (CA) (**a-c**) and femoral arteries (FA) (**d-f**) of wild-type (WT) and PACAP deficient (KO) mice. Data are expressed as means ± SEM (n = 5-6/group). * p < 0.05 WT male *vs*. female, ^#^ p < 0.05 KO male *vs*. female. Achieved force is normalized to maximal contraction induced by KCl (60mM) for easier comparison.
(TIF)

S2 Fig Protein expression of PAC1, VPAC1 and VPAC2 in carotid arteries (CA) and femoral arteries (FA) of wild type (WT) and PACAP deficient (KO) mice. Representative data of two independent animal samples. *p < 0.05 vs. control (WT mice).
(TIF)

S3 Fig Vasomotor ability to KCl-induced contraction in carotid (CA) and femoral (FA) arteries of wild tpye (WT) and PACAP deficient (KO) mice (**a**). Isometric force is normalized to the maximal achievable contraction with 60mM KCl. Vasorelaxation properties were tested with achetylcholine (Ach) (left panel) and sodium nitroprussid (SNP) (right panel) in both WT (**b**) and KO (**c**) mice of carotid (on the left side) and femoral arteries (on the right side). Values are expressed as means ± SEM (n = 6/group). * p < 0.05 WT *vs*. KO mice.
(TIF)

S4 Fig Vasomotor effects of carotid (CA) and femoral (FA) arteries of WT and PACAP KO mice (**a-d**) in response to PACAP1-38, PACAP1-27 and VIP during four stages of the female reproductive cycle in mice: proestrus (indicated by circles), estrus (indicated by square), metestrus (indicated by diamonds) and diestrus (indicated by triangles). There is no significant difference between stages of the estrus cycle.
(TIF)

## Abbreviations

PACAP: pituitary adenylate cyclase-activating polypeptide
PACAP^+/+^: mouse with functional PACAP gene (WT)
PACAP^-/-^: mouse with knockout PACAP gene (KO)
PACAP1-38: 38 amino acid form of PACAP
PACAP1-27: 27 amino acid form of PACAP
VIP: vasoactive intestinal peptide
PAC1R: PACAP type I receptor
VPAC1R: vasoactive intestinal peptide receptor 1
VPAC2R: vasoactive intestinal peptide receptor 2
M65: selective PAC1R antagonist
VIP6-28: selective VPAC1R antagonist
Ala^11.22.28^VIP: selective VPAC1R agonist
Bay55-9837: selective VPAC2R agonist
Ach: Acetylcholine
SNP: Sodium nitroprusside
cAMP: cyclic adenosine monophosphate
PKA: protein kinase A
