# Supplementary figures and images for: VPAC1 receptors play a dominant role in PACAP-induced vasorelaxation in female mice
Source: PLoS One. 2019 Jan 25;14(1):e0211433. doi: 10.1371/journal.pone.0211433 (PMC6347420; doi:10.1371/journal.pone.0211433)

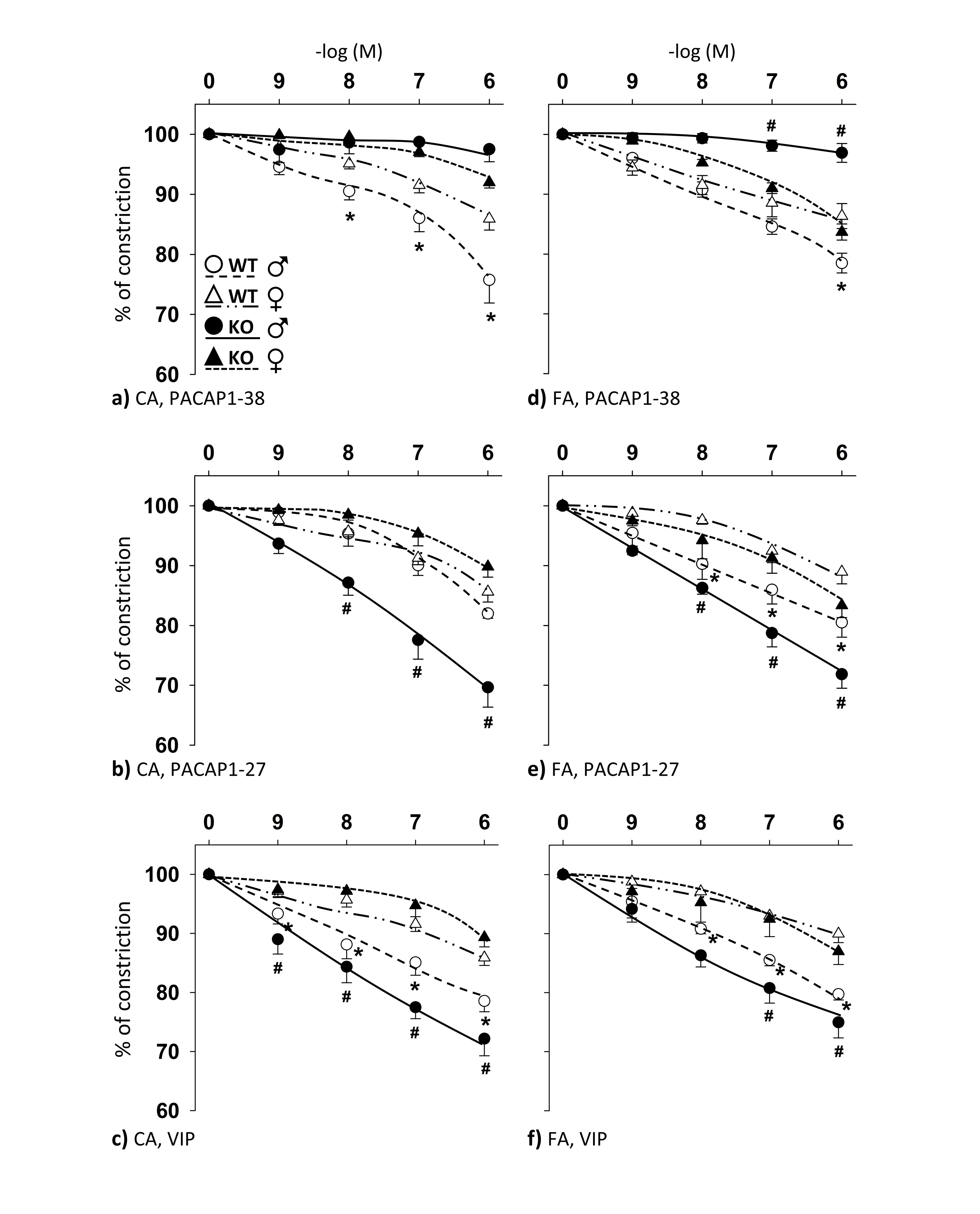

Supplement: S1 Fig — Gender-dependent differences of cumulative dose-dependent administration of PACAP1-38 (a and d); PACAP1-27 (b and e) and VIP (c and f) on the vasomotor response in carotid arteries (CA) (a-c) and femoral arteries (FA) (d-f) of wild-type (WT) and PACAP deficient (KO) mice. Data are expressed as means ± SEM (n = 5-6/group). * p < 0.05 WT male vs. female, # p < 0.05 KO male vs. female. Achieved force is normalized to maximal contraction induced by KCl (60mM) for easier comparison. (TIF) [file pone.0211433.s001.tif]

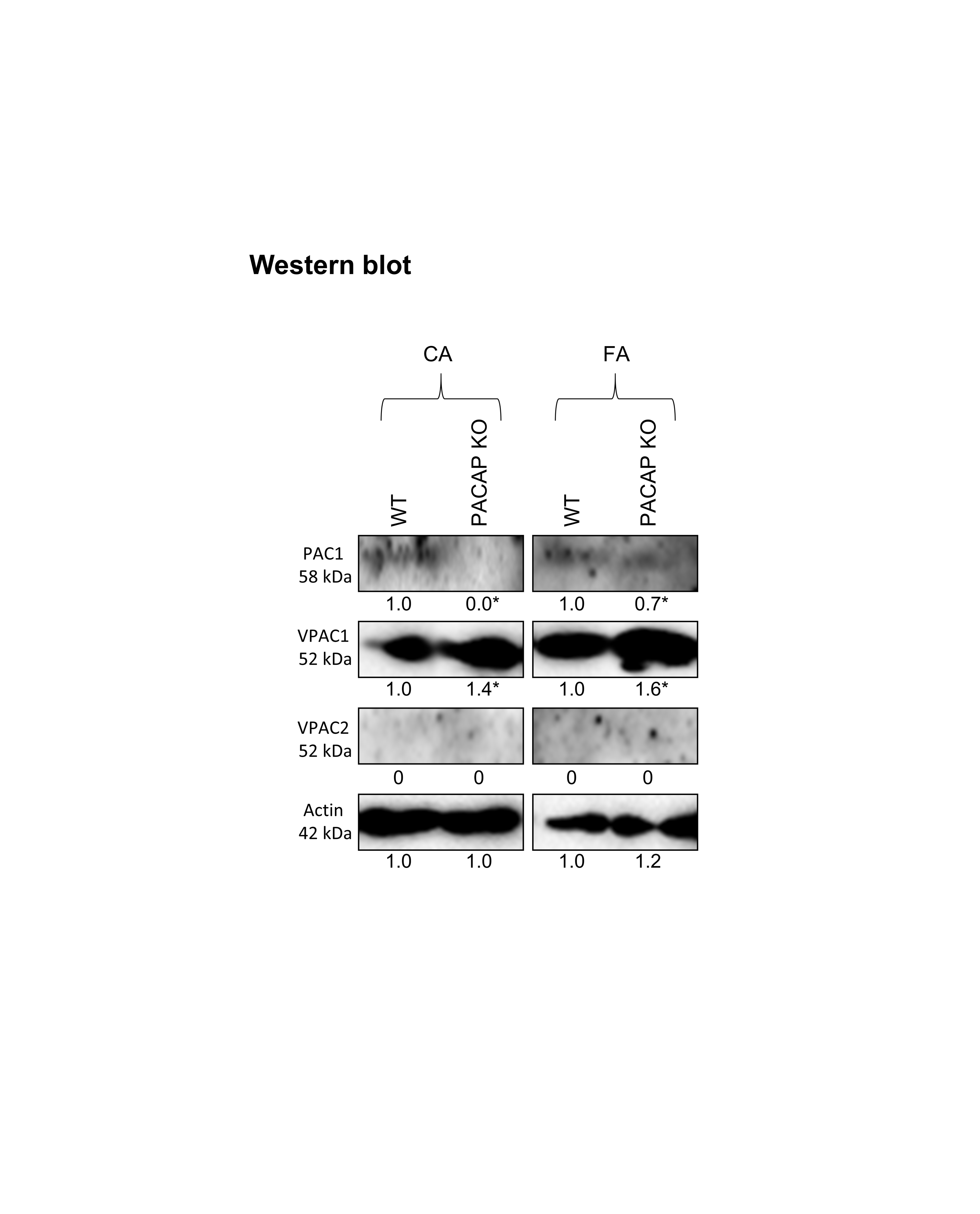

Supplement: S2 Fig — Representative data of two independent animal samples. *p < 0.05 vs. control (WT mice). (TIF) [file pone.0211433.s002.tif]

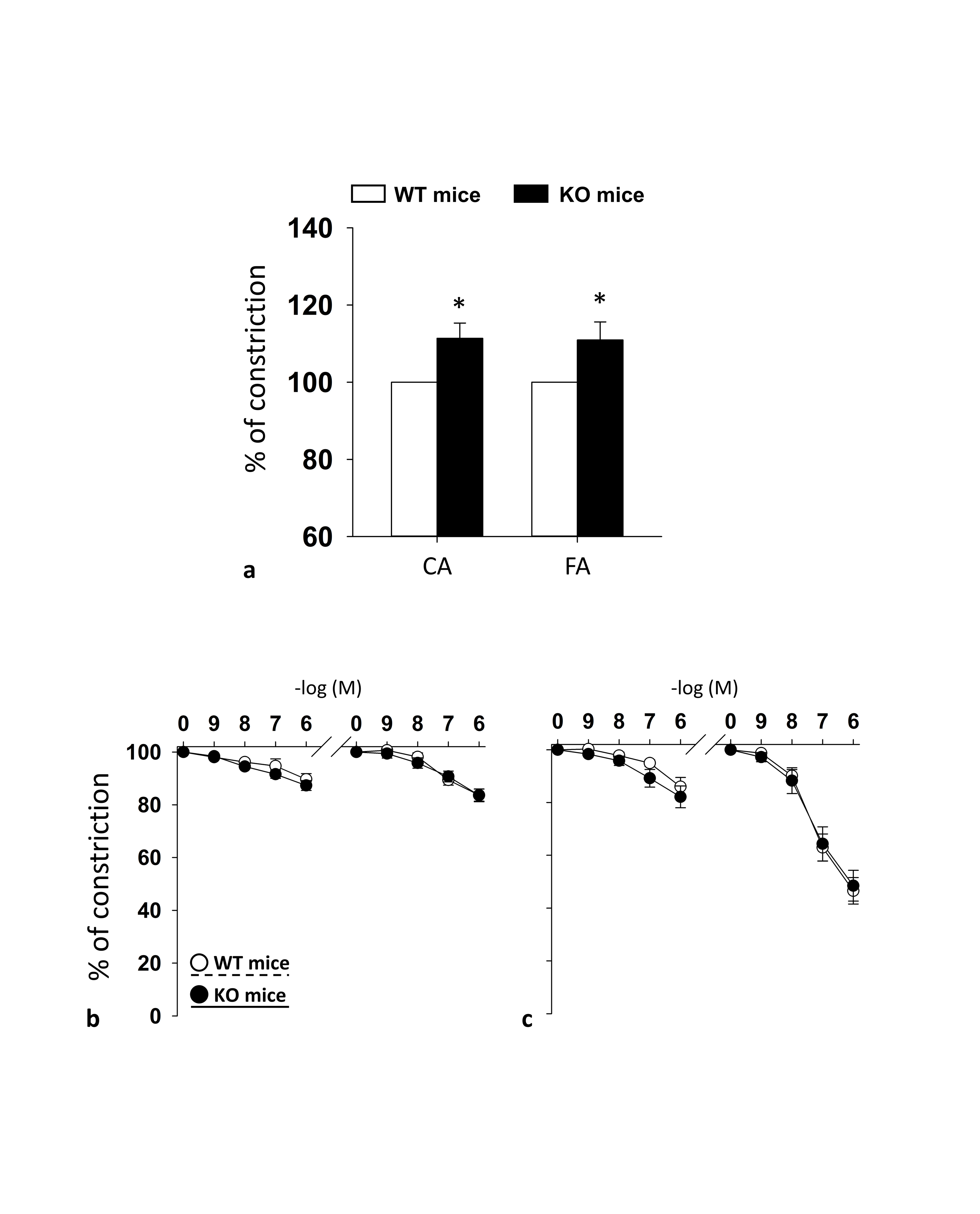

Supplement: S3 Fig — Vasomotor ability to KCl-induced contraction in carotid (CA) and femoral (FA) arteries of wild tpye (WT) and PACAP deficient (KO) mice (a). Isometric force is normalized to the maximal achievable contraction with 60mM KCl. Vasorelaxation properties were tested with achetylcholine (Ach) (left panel) and sodium nitroprussid (SNP) (right panel) in both WT (b) and KO (c) mice of carotid (on the left side) and femoral arteries (on the right side). Values are expressed as means ± SEM (n = 6/group). * p < 0.05 WT vs. KO mice. (TIF) [file pone.0211433.s003.tif]

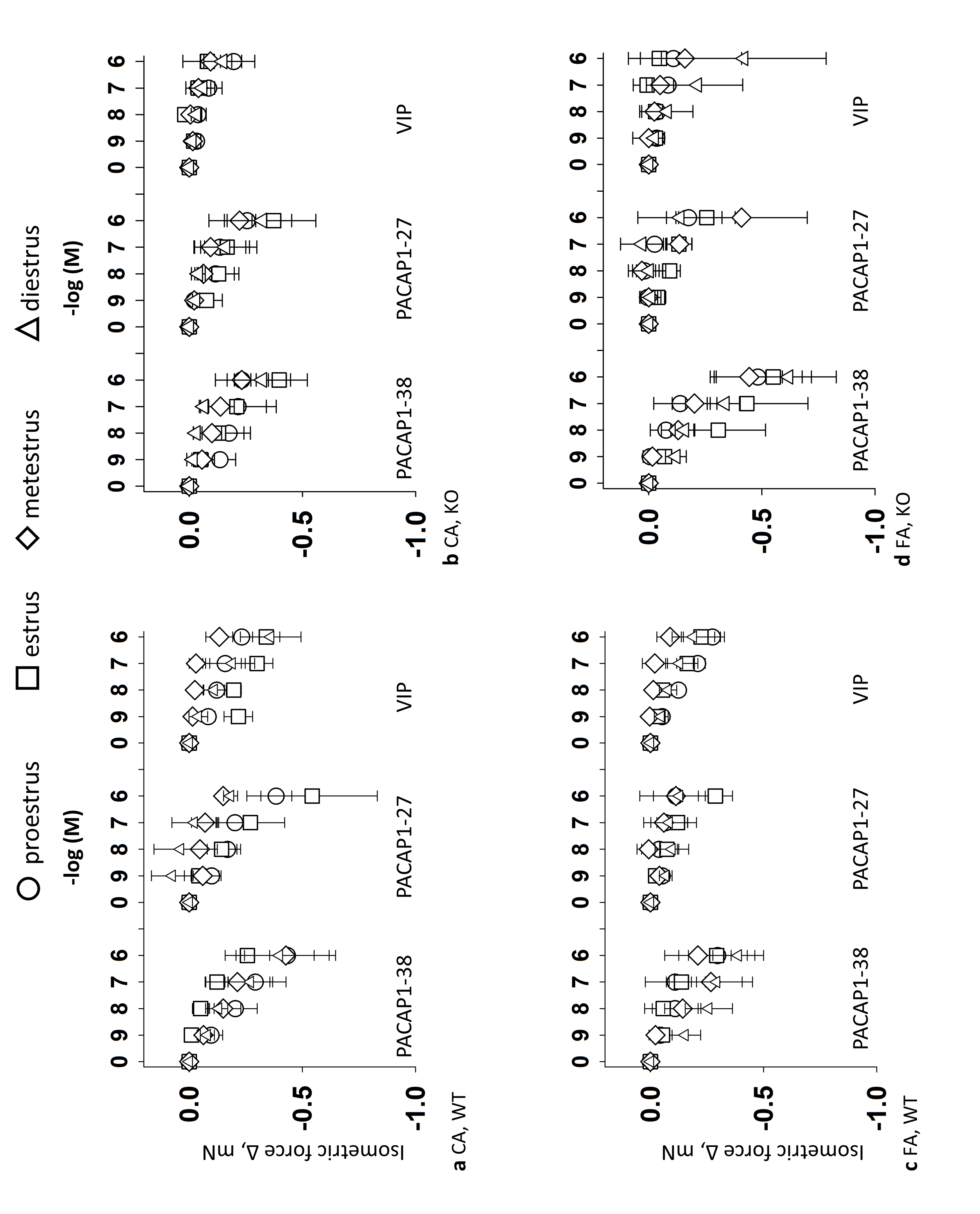

Supplement: S4 Fig — Vasomotor effects of carotid (CA) and femoral (FA) arteries of WT and PACAP KO mice (a-d) in response to PACAP1-38, PACAP1-27 and VIP during four stages of the female reproductive cycle in mice: proestrus (indicated by circles), estrus (indicated by square), metestrus (indicated by diamonds) and diestrus (indicated by triangles). There is no significant difference between stages of the estrus cycle. (TIF) [file pone.0211433.s004.tif]
